# Correction: Patch test results in paediatric patients with atopic dermatitis in Laos

**DOI:** 10.1371/journal.pone.0236215

**Published:** 2020-07-09

**Authors:** Catriona I. Wootton, Mong K. Sodaly, Somxay X. Billamay, John S. C. English, Mayfong Mayxay

The fifth author's name is incorrect. The correct name is: Mayfong Mayxay. The correct citation is: Wootton CI, Sodaly MK, Billamay SX, English JSC, Mayxay M (2020) Patch test results in paediatric patients with atopic dermatitis in Laos. PLoS ONE 15(4): e0231455. https://doi.org/10.1371/journal.pone.0231455
