# Supplementary material for: Patch test results in paediatric patients with atopic dermatitis in Laos
Source: PLoS One. 2020 Apr 14;15(4):e0231455. doi: 10.1371/journal.pone.0231455 (PMC7156065; doi:10.1371/journal.pone.0231455)
Supplement: S1 Fig — (DOCX) [file pone.0231455.s001.docx]

50 AD patients known to clinic contacted and asked if wanted to participate

30 newly diagnosed AD patients asked if wanted to participate

24 patients willing to participate

26 patients willing to participate

All 50 patients consented to take part completed the study
