# Supplementary material for: Patch test results in paediatric patients with atopic dermatitis in Laos
Source: PLoS One. 2020 Apr 14;15(4):e0231455. doi: 10.1371/journal.pone.0231455 (PMC7156065; doi:10.1371/journal.pone.0231455)
Supplement: Attachment — Submitted filename: Response to reviewers Paeds PT PONE-D-19-12757.docx [file pone.0231455.s004.docx]

Response to reviewers

PONE-D-19-12757

(Author replies in red)

Please include a flowchart of the tested patients and any information about possible selection process

A flowchart detailing the patient selection process has now been included.

Journal Requirements:

When submitting your revision, we need you to address these additional requirements.

1. Please ensure that your manuscript meets PLOS ONE's style requirements, including those for file naming. The PLOS ONE style templates can be found at

<http://www.journals.plos.org/plosone/s/file?id=wjVg/PLOSOne_formatting_sample_main_body.pdf> and <http://www.journals.plos.org/plosone/s/file?id=ba62/PLOSOne_formatting_sample_title_authors_affiliations.pdf>

The adjustments to meet PLOS ONE’s style have been made.

2. Please specify in your ethics statement: 1) whether the ethics committee approved the verbal/oral consent procedure, 2) why written consent could not be obtained, and 3) how verbal/oral consent was recorded. Please also state whether consent was suitably informed.

The text has now been adjusted to read: *“The process of patch testing was explained and images of patch tests being performed were also shown to the patient and their family and verbal consent given. Verbal consent was deemed appropriate for the study as this is standard practice for patch testing in the author’s experience and consent was recorded on the study proforma at each visit.”*

3. Please include in your Methods section the date ranges over which you recruited participants to this study.

The following has been added to the text: “*This study ran from August 2017 to January 2018”.*

Reviewers' comments:

Reviewer's Responses to Questions

**Comments to the Author**

5. Review Comments to the Author

Reviewer #1: This paper is an original work that I believe, for the first time, calls attention on contact sensitivity in a paediatric population with Atopic Dermatitis in Laos. This paper is methodologically accurate and well written.
The reference section is appropriate and sufficiently up-to date, but the references are not always correctly cited in the discussion: as a matter of fact, Boonchai et al is a reference 17 and not 14, and How et al is reference 16 and not 13.

Thank you for highlighting the error with reference numbering, this has now been corrected.

The very high incidence of contact sensitization to gold is somehow a bit surprising. It would be interesting to know if the reaction had a dynamic pattern “in crescendo” in order to better clarify if the reactions where true “allergic” reactions.

All of the reactions to gold were crescendo in nature and the results of this study echo very similar findings in other studies performed in South East Asia and the Middle East as well as the study run in conjunction with this, looking at medical students with evidence of contact dermatitis. We agree it is certainly an interesting finding but we are confident these were true positives.

The authors state: “PPD is not often reported as a common allergen in the paediatric population….” and: “the majority (80%) of our participant where under the age of 5 years. As a result, direct comparison with other studies looking at ACD in the paediatric AD population may not be feasible...”
About these topics, I suggest reading also:

1. Belloni Fortina A, Romano I, Peserico A, Eichenfield LF Contact sensitization in very young children. J Am Acad Dermatol. 2011 Oct;65(4):772-779.
2. Belloni Fortina A, Cooper SM, Spiewak R, Fontana E, Schnuch A, Uter W. Patch test results in children and adolescents across Europe. Analysis of the ESSCA Network 2002-2010. Pediatr Allergy Immunol. 2015 Aug;26(5):446-55
3. Belloni Fortina A, Fontana E, Peserico A. Contact Sensitization in Children: A Retrospective Study of 2,614 Children from a Single Center. Pediatr Dermatol. 2016 Jul;33(4):399-404.

Thank you for suggesting these papers, they certainly make interesting reading. The following has been added to the manuscript: “*Interestingly, Belloni Fortina et al (2015) considered paediatric patch test results across Europe and found that the highest sensitization rate was in the age group of 1-5 years (45.3% compared to 33.3% between ages 6-12 years). In a further study, Belloni Fortine et al (2011) looked at contact sensitization in children aged between 3 and 36 months and found an even higher rate of positive reactions (62.3%), this was in a cohort of children both with and without atopic dermatitis but all suspected of having contact dermatitis. Both of these papers highlight that contact sensitization is high in the very young, and the results of our study fit with this data.”*

As regards PPD in very young children that have not been temporary tattooed see in particular Belloni Fortina et al - JAAD 2011 page 776.

The section on PPD in this paper is certainly very interesting; in our cohort, those children testing positive to PPD had a very clear history of repeated exposure to parental hair dye so we are fairly confident that this is the route of exposure, especially as it is common practice to share a bed in these communities.

Furthermore, the authors state: “it would also have been interesting to patch test healthy controls.”. I have some doubts from an ethical point of view of the feasibility of patch testing healthy controls.

Yes, given the risk of sensitization this is a very valid point. We have changed the text to read: “*This was not done in this study due to the limited number of patch tests available and it was felt that children with AD may yield more positive results than healthy controls, in addition it is important to consider the risk of sensitization.”*

Reviewer #2: 1) the authors could't define the relevance of the responses to the Patch test, then the best Title is: Patch test results in paediatric patients with atopic dermatitis in Laos.

The title has been changed as you suggest to: *Patch test results in paediatric patients with atopic dermatitis in Laos*

2) its necessary to discuss the use of TRUE test in pediatric patients. Its no so common to use this kind of patch test in this group. I suggest the paper: Safety and efficacy evaluation of TRUE TEST panels 1.1, 2.1, and 3.1 in children and adolescents. Dermatitis. 2011 Jul-Aug;22(4):204-10.

Thank you for suggesting this paper. We agree that it is not so common to use this type of patch test in children but given the location (temperature, humidity, lack of space, etc) and nature of the study, the only feasible way to perform the patch testing was to use prepared strip such as the TRUE tests. The study you recommended found no increased risk of adverse events in the paediatric population tested using TRUE test and our experience also did not highlight any concerns using this system to perform patch tests in paediatric patients.
